# Fully Biobased Photothermal Films and Coatings for Indoor Ultraviolet Radiation and Heat Management

**DOI:** 10.1021/acsami.2c00718

**Published:** 2022-03-01

**Authors:** Jinrong Liu, Adrian Moreno, Jian Chang, Mohammad Morsali, Jiayin Yuan, Mika H. Sipponen

**Affiliations:** Department of Materials and Environmental Chemistry, Stockholm University, Svante Arrhenius väg 16C, SE-106 91 Stockholm, Sweden

**Keywords:** photothermal, light management, passive cooling, fully biofilm, lignin

## Abstract

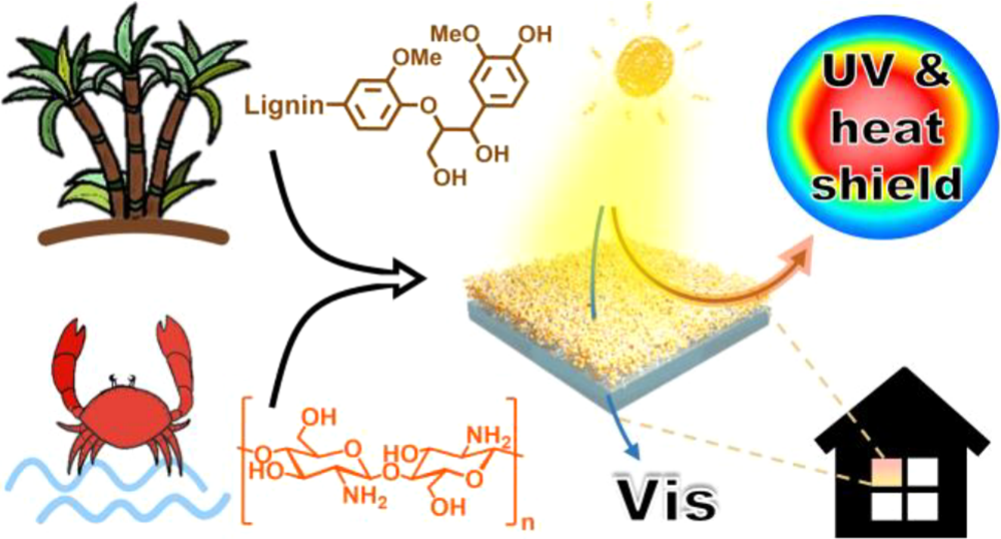

Sustainable materials are needed to mitigate against the increase in energy consumption resulting from population growth and urbanization. Here, we report fully biobased nanocomposite films and coatings that display efficient photothermal activity and selective absorption of ultraviolet (UV) radiation. The nanocomposites with 20 wt % of lignin nanoparticles (LNPs) embedded in a chitosan matrix displayed an efficient UV blocking of 97% at 400 nm along with solar energy-harvesting properties. The reflectance spectra of the nanocomposite films revealed the importance of well-dispersed nanoparticles in the matrix to achieve efficient UV-blocking properties. Finally, yet importantly, we demonstrate the nanocomposites with 20 wt % LNPs as photothermal glass coatings for passive cooling of indoor temperature by simply tailoring the coating thickness. Under simulated solar irradiation of 100 mW/cm^2^, the 20 μm coating achieved a 58% decrease in the temperature increment in comparison to the system with uncoated glass. These renewable nanocomposite films and coatings are highly promising sustainable solutions to facilitate indoor thermal management and improve human health and well-being.

## 1 Introduction

Energy consumption is constantly rising because of urbanization and population growth, and global climate change increases the need for better heating, cooling, and lighting systems.^1−5^ In this context, great efforts have been made to develop more energy-efficient, ideally sustainable materials with switchable optical properties for maintaining indoor temperatures.^6−8^ Recently, “smart” polymer coatings^9−11^ and films^12,13^ have emerged as energy-efficient materials offering improved photothermal properties in comparison to traditional glass windows and ceilings to control the heat flow across the material. Such films and coatings are also expected to carry suitable optical properties in energy-saving windows, including good transmittance in the visible-light region and selective absorption of ultraviolet (UV) radiation for protecting human health and materials from UV-initiated degradation. The high energy of UV radiation (corresponding to wavelengths of 200–400 nm) can damage DNA,^14^ trigger food deterioration,^15^ and fade colors in historical artworks.^16^ In addition to photothermal and optical performances, it is also important to ensure sufficient mechanical properties and derive materials from renewable natural polymers. For example, transparent wood materials were reported by removing natural lignin from wood.^6,17^ However, scale-up of these materials faces significant challenges stemming from the use of harmful chemicals during the extraction and bleaching steps.

Learning from nature, one can appreciate lignin as an important component that reinforces and protects plant tissues from UV radiation.^18^ Unfortunately, industrial lignin is mainly treated as a waste or byproduct from the pulping industry and primarily burned away to gain heat because of the shortage of commercially feasible value-added products.^19^ Therefore, more efforts are needed to develop materials that harness the rich chemical structure and composition available in technical lignin. The abundant chromophore groups that contribute to UV absorption in lignin include conjugated phenolics, ketones, quinoid structures, and intramolecular hydrogen bonds. Based on the understanding of radiation absorption by lignin, the photothermal conversion of lignin has been recently reported.^20−22^ For example, Chen’s group^20^ showed that lignin can carry out photothermal conversion and serve to power a thermoelectric generator. However, lignin’s dark color and its compromising impact on visible transparency are still big challenges among lignin-based materials.

Lignin–polymer composites are potential materials for absorbing solar energy without sacrificing visible transparency. However, the main challenge lies in the immiscibility of lignin with polymers because of the poor solubility and interactions of lignin within polymeric matrix matrices.^23−29^ In this context, lignin nanoparticles (LNPs) have emerged as an alternative to maintain the mechanical strength of lignin-based polymeric materials.^30−34^ Several transparent lignin–polymer composites with lignin content below 20 wt % have been reported, such as lignin–poly(vinyl alcohol) (PVA),^35,36^ lignin–polystyrene, and lignin–poly(butyl methacrylate).^37^ However, their photothermal properties have been so far ignored.

The outstanding challenge is to fabricate photothermal composite coatings and films that are made of lignin in combination with only natural polymers, which allow transmittance of visible light. Chitosan (Chi) is the deacetylated product of chitin that has many unique properties, including biocompatibility, biodegradability, nontoxicity, antimicrobial, and excellent film forming properties.^38,39^ The combination of lignin and Chi or chitin^40^ has attracted renewed interest because of their electrostatically favored molecular interactions. For instance, LNP-Chi nanocomposites show good emulsion stabilization and thermal stability.^41−43^ The water absorption by Chi films can be decreased by chemical modification,^44^ crosslinking agents,^45^ or hydrophobic spray coating.^46^ These properties make Chi a potential candidate for the development of new photothermal nanocomposites with lignin.

Here, we demonstrate LNP–biopolymer hybrid films and coatings using Chi as a continuous matrix. We show free-standing and uniform lignin nanoparticle-chitosan (LNP-Chi) films with 10–40 wt % of lignin. The advantage of LNPs in improving the stability of the composites is clarified by comparing the morphologies and mechanical performances of films prepared from well-defined spherical lignin nanoparticles mixed with chitosan solution (LNP-Chi films) to those containing in situ lignin particles precipitated in the confined Chi matrix during the film casting process (SL-Chi films). The in situ formation of lignin particles on chitin nanofibers has been reported by similar methods such as solvent shifting.^47^ However, in this work LNPs in the Chi matrix show more uniform dispersion than the in situ lignin particles. The mechanical properties of LNP-Chi films, therefore, are higher than those of SL-Chi films. Based on the favorable mechanical properties, we display optical and photothermal properties of LNP-Chi films and demonstrate LNPs-Chi as transparent photothermal coatings for potential use in energy-efficient building materials.

## 2 Experimental Section

### 2.1 Chemicals and Materials

Soda lignin (SL; PROTOBIND 2400, GreenValue LLC) was used to prepare all lignin materials in this work. ^31^P NMR spectra of SL were recorded following a previously described methodology.^48^ Briefly, a 90° pulse angle, inversed gated proton decoupling, and a delay time (D1) of 10 s were used. For the analysis, 256 scans with a total runtime of 30 min were used for each sample. Three replicated experiments were conducted, and the mean value of one standard deviation is reported. All the chemicals and solvents were purchased from Sigma-Aldrich, Fischer, and VWR and were used as received.

### 2.2 Preparation of the LNP Dispersion

LNPs were made according to the solvent polarity shifting method^33^ using SL as a resource. In this work, 2.5 g of SL was added into a mixed solvent containing 50 g deionized water and 150 g acetone. After stirring for 3 h, the SL solution (acetone: water, 3:1 w/w) was filtered twice to remove insoluble impurities. Deionized water (600 mL) was added to the lignin solution to cause precipitation of lignin and formation of an aqueous dispersion of LNPs by decreasing the solvation ability for lignin. During this process, deionized water was poured within 5 s to the lignin solution that was subjected to magnetic stirring at 600 rpm. Acetone in the formed LNP dispersion was removed using a rotary evaporator. In particular, the formed LNP dispersion (with acetone) was placed in an evaporation flask, fitted to the inlet of the vacuum system with a vacuum of 300 mbar and heated with a water bath set at a temperature of 40 °C, and rotated at a rotation rate of 90 rpm. The evaporation process was performed by decreasing the pressure step-by-step until 40 mbar was reached. Then the evaporation was continued for an additional 30 min to evaporate acetone completely. After removing acetone, the final LNP dispersion was available for preparing film and coating materials. The concentration of LNPs in the final dispersion was 4 g/L (0.42 wt %).

### 2.3 Preparation of LNP-Chi Films and SL-Chi Films

Hybrid solutions with different contents of LNPs (10–40 wt %) and Chi (90–60 wt %) were prepared by adding the LNP dispersion (4 g/L) dropwise to a stirred solution of 1 wt % Chi in 0.1 M acetic acid. Then LNPs-Chi films (LNPs_x_-Chi_y_) were formed by casting the dispersion mixture on polypropylene Petri dishes. For example, 1.5 mL LNP dispersion was added to 2.4 g Chi solution (1 wt %) and then transferred to a plastic dish, after evaporating at room temperature (RT) for 3 days, the film LNPs_20_-Chi_80_ was formed. Here we label the samples with wt %; for example, LNPs_20_-Chi_80_ means nanocomposites containing 20 wt % LNPs and 80 wt % Chi. For comparison, SL-Chi films were prepared by the same method with LNPs-Chi films except using the SL solution (acetone: water, 3:1 w/w) instead of an aqueous dispersion of LNPs.

### 2.4 Preparation of Coated Glass

The nanocomposite coatings on glass were prepared by depositing LNPs_20_-Chi_80_ on glass dishes and drying at 110 °C for 6 h. The thickness of the coating layer was assumed from the amount deposited. For example, by cutting coated glass and examining its cross section, we found that 3.9 g LNPs_20_-Chi_80_ hybrid solution formed 5 μm coating layer. Then, the thicknesses of the glass coatings resulting from casting of 7.8, 11.7, and 15.6 g of the nanocomposite formulation were assumed to be 10, 15, and 20 μm, respectively.

### 2.5 Characterization of LNP-Chi and SL-Chi Composites

A Zetasizer Nano ZS (Malvern, UK) was used to measure particle size distributions of LNPs by dynamic light scattering (DLS) and zeta potential by electrophoretic mobility using a dip cell probe. Scanning electron microscopy (SEM) images of films and coatings were recorded using a JSM-7000F with an accelerating voltage of 1–5 kV. The samples were coated with Au before observing by SEM. Transmission electron microscopy (TEM) images of LNPs and a LNP-Chi composite were recorded using a JEM-2100LaB6 with an accelerating voltage of 120 kV. The samples were prepared by depositing and drying dispersions on Cu grids. Atomic force microscopy (AFM) analysis was performed on a MultiMode AFM Nanoscope V (Veeco Instruments, USA). The images were obtained in ScanAsyst mode under ambient air conditions with SCANASYST-AIR probes (Bruker). Uniaxial mechanical properties of films were evaluated using an Instron 5960 universal testing machine (Instron, USA) with a 100 N load cell. The samples were cut to a rectangular shape with dimensions of 3 × 25 mm and conditioned in a room with constant 50% relative humidity at 23 °C overnight before testing. The thickness of films was measured using an electronic outside micrometer (Schut Geometrical Metrology). The samples were measured using a gauge length of 1 cm and a strain rate of 4 mm min^–1^. A UV–Vis spectrophotometer (Agilent Cary 5000 UV–Vis–NIR) was used to determine the optical properties of films and coatings. The in-line transmittance spectra of films and coatings were recorded in the range of 200–800 nm with air as background. The reflectance of films was recorded with the praying mantis diffuse reflectance accessory in the range of 200–900 nm with a specialized fluorine-based polymer as reference. Photothermal properties of films and coatings were measured using a thermal camera (Testo 872) and temperature data logger (Testo 175 T3). The temperature changes and the infrared images of films and coatings were recorded. During the measurement, samples were irradiated under controlled 1 sun irradiation (100 mW/cm^2^) with a light-emitting diode (LED) lamp ABA LED Solar simulator (Newport LSH-7320) as the light source.

## 3 Results and Discussion

### 3.1 Fabrication of Transparent LNP-Chi Films

**Figure 1:**
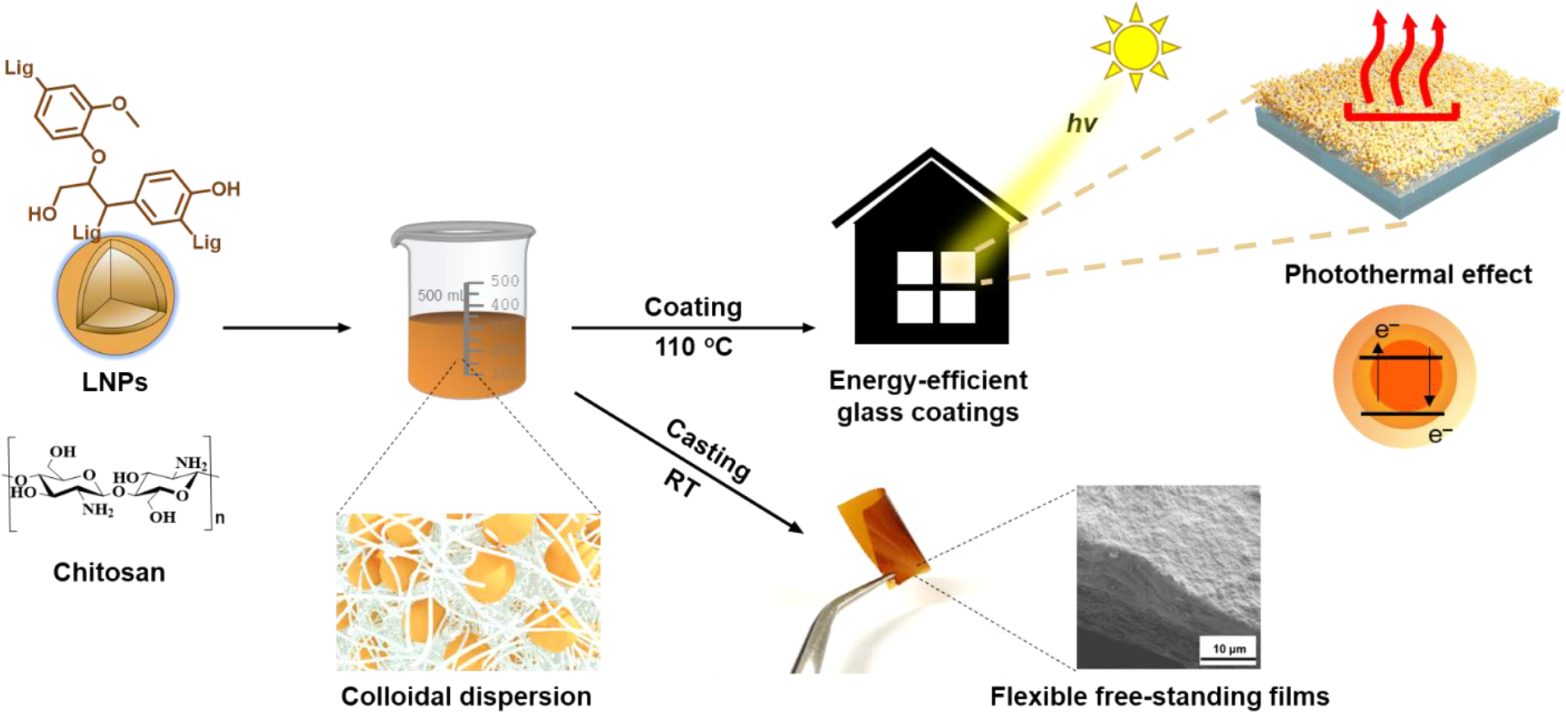
General process scheme for the preparation and application of LNP-Chi films and coatings. LNPs, Chi , and RT.

The mechanical and optical properties of nanocomposite materials depend on colloidal stability of the dispersion used in their preparation, which according to the Derjaguin–Landau–Vervey–Overbeek theory is controlled by attractive and repulsive interactions.^37,49−51^ Here, we prepared composite materials using LNPs and Chi that are known to interact via attractive electrostatic forces and hydrogen bonding.^33,41^ As shown in Figure 1, our approach to prepare films and coatings is based on a combination of LNPs (prepared via a solvent-exchange methodology in Supporting Information Figure S1) and Chi *via* two steps: (i) preparation of the LNP-Chi colloidal dispersion and (ii) using the casting method to prepare LNPs-Chi as free-standing films, or coatings using glass as a substrate. As final target application, we demonstrate LNPs-Chi as transparent coatings for passive cooling of indoor temperature because of its ability to absorb solar energy and block UV radiation, which ultimately would be promising candidates for energy-efficient building applications.

**Figure 2:**
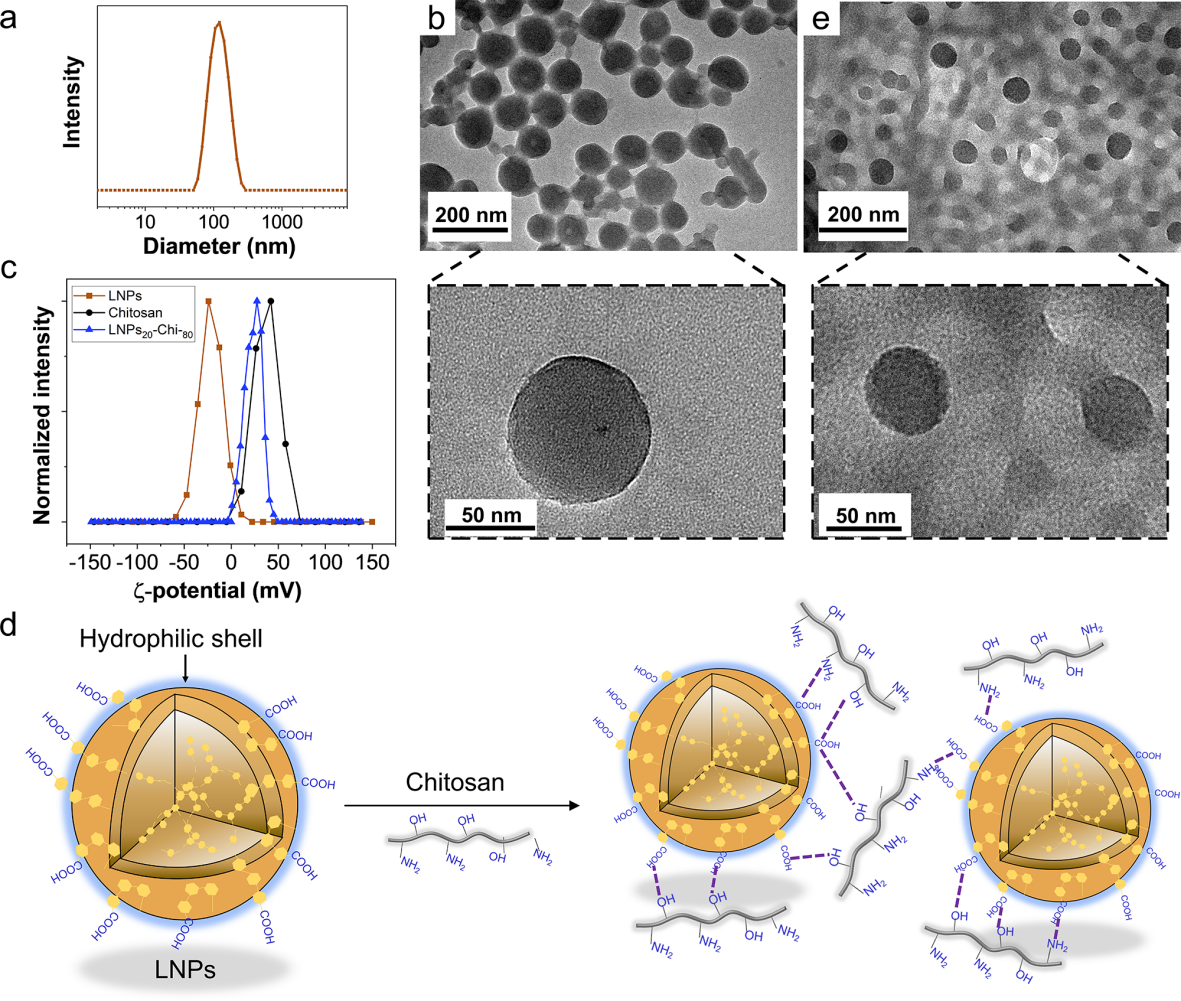
Characterization of LNPs and the LNPs_20_-Chi_80_ composite. (a) Diameter distribution of LNPs from DLS measurement. (b) Representative TEM image of LNPs.(c) Zeta (ζ) potential of the aqueous dispersion of LNPs, Chi solution, and aqueous dispersion of LNPs_20_-Chi_80_. (d) Schematic illustration of interaction forces between LNPs and Chi. (e) Representative TEM image of LNPs_20_-Chi_80_.

LNPs prepared by the solvent-exchange method exhibited a particle diameter of 120 nm, with a low polydispersity index of 0.08 determined by DLS (Figure 2a). These results are in agreement with TEM analysis of LNPs that confirmed their spherical particle geometry (Figure 2b). By immersing the aqueous dispersion of LNPs (ζ-potential −24 mV) in Chi solution (ζ-potential +42 mV), the surfaces of the particles became saturated with Chi chains. Previous studies have demonstrated that colloidally stable LNPs with cationic net charge can be produced by adsorbing 5–10 wt % of Chi on LNPs.^33,41,52^ Here, Chi was used in excess, and the overall system had a cationic ζ-potential. The ζ-potential of the LNPs_20_-Chi_80_ dispersion was +27 mV, and no aggregation was observed (Figure 2c and Supporting Information Figure S2). The reduction of ζ-potential suggests ion exchange at the protonated primary amines of Chi by anionic groups of LNPs, mainly its surface-enriched carboxylate groups.^53^ As shown in Supporting Information Figure S3 and Table S1, the total phenolic OH, carboxylic OH, and aliphatic OH contents of SL were quantitatively analyzed by ^31^P NMR spectroscopy and found to be almost similar as previously reported for another type of SL.^54^ Therefore, the Coulomb force interactions in the hybrid colloid improve the stability of the dispersion as shown in Figure 2d. An equally important intermolecular force is the hydrogen bonding between the components. After evaporation of the LNP-Chi dispersion, films with thicknesses of approximately 20 μm were formed. When deposited as a thin film on a carbon grid, TEM micrographs revealed individual nanoparticles embedded in the Chi matrix without detectable aggregation, as also indicated by the observed stability of the mixed colloidal dispersion (Figure 2e).

### 3.2 Mechanical Properties of LNP-Chi Films

Lignin is typically brittle and difficult to disperse in polymeric solutions. This has limited the development of composites with appropriate morphological and made it hard to obtain materials with sufficient mechanical properties. The lignin content of polymeric composites is often below 10 wt %,^30,31,55^ which places severe limits on the benefits brought from the intrinsic functionality of lignin, such as UV blocking, antibacterial, fluorescence, and so on. However, LNPs in comparison to bulk lignin display a higher specific surface area enriched with hydrophilic functional groups. This may not only stabilize, but also improve the mechanical properties by promoting the noncovalent interactions of lignin within the polymeric matrix, while also acting as sacrificial noncovalent interactions.^37,53^

**Figure 3:**
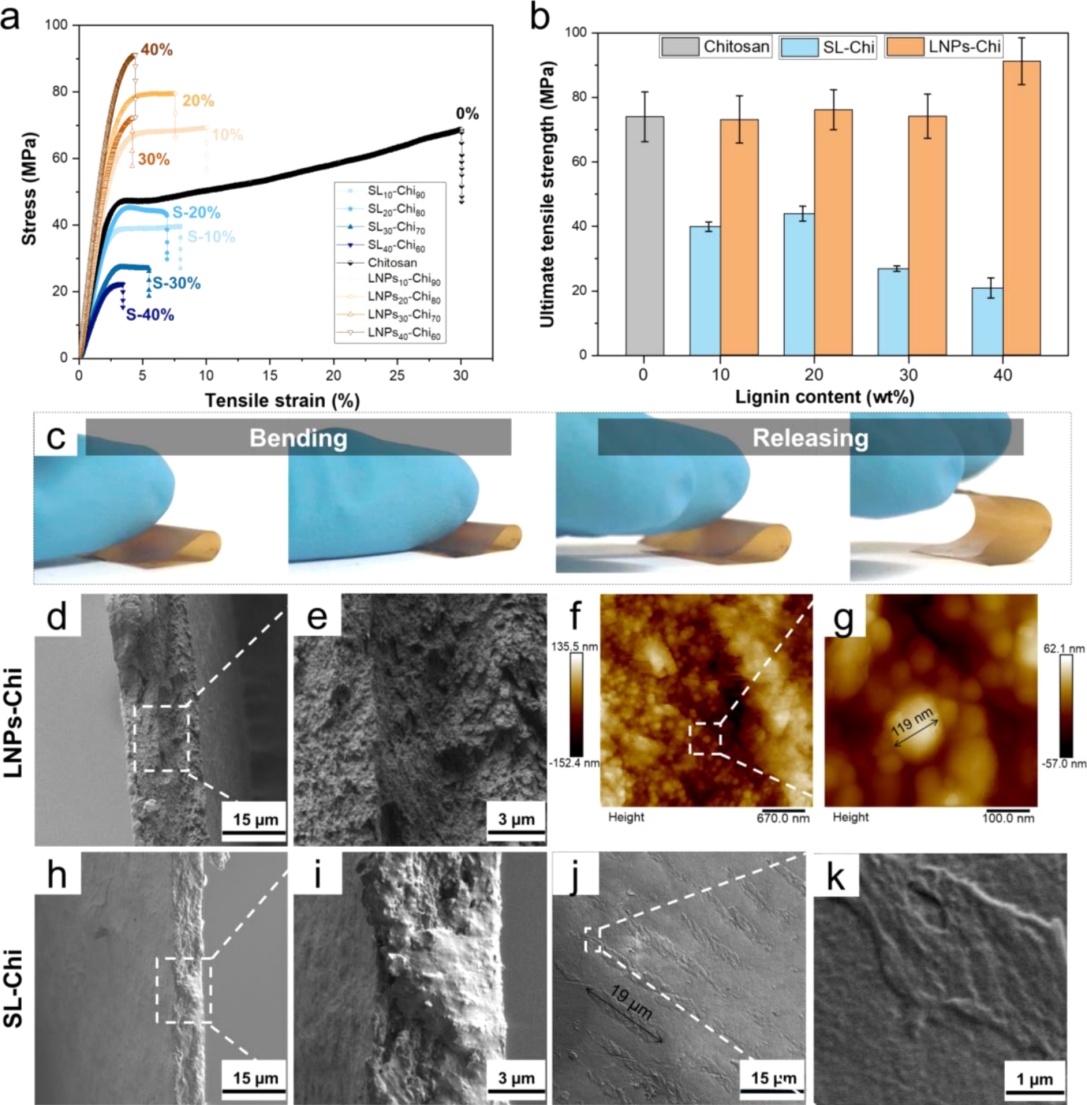
Mechanical properties and morphological comparison of LNP-Chi and SL-Chi films. (a) Tensile stress–strain curves and (b) UTS of pure Chi (0%), LNP-Chi films, and SL-Chi films (10–40 wt % lignin content). (c) Digital photos of LNPs_20_-Chi_80_ recorded in the bending and releasing process. SEM images of the fracture cross section of (d,e) LNPs_40_-Chi_60_ and (h,i) SL_40_-Chi_60_, and surface morphology of (j,k) SL_40_-Chi_60_. (f,g) AFM images of the surface of LNPs_40_-Chi_60_. The error bars represent one standard deviation from the mean values (*n* = 3).

To elucidate the effects of the particle size and morphology, we compared the tensile strength of LNP-Chi and SL-Chi films to that of pristine Chi film. All LNP-Chi composition films showed similar or improved ultimate tensile strength (UTS) compared to that of the pristine Chi film (Figure 3a,b). For example, the UTS of LNPs_40_-Chi_60_ was 91 MPa, compared to 73 MPa for the Chi film. Pristine Chi films displayed an initial elastic behavior below 3% strain followed by plastic deformation (Figure 3a, black line). The tensile strength of LNP-Chi films with up to 40 wt % lignin is higher than that of lignin-Chi films with 1 wt % of unfractionated kraft lignin (UTS: 80 MPa) or organosolv lignin (UTS: 75 MPa),^56^ but lower than that of a blend film of lignosulfonate with synthetic polyamide–epichlorohydrin polycation (UTS: 102 MPa)^57^ (Supporting Information Table S2). Despite its high lignin content, LNPs_20_-Chi_80_ can be folded without breaking and recovered to the original shape after releasing the force (Figure 3c), which is of practical importance for applications in which the films need to resist physical forces from assembly and operation.

To compare the impact of lignin morphology on mechanical properties, we tested tensile strength of SL-Chi films (containing spatially confined precipitated lignin in the Chi matrix, see Supporting Information Figure S4) with a lignin content of 10–40 wt %. Figure 3a shows that SL-Chi films had a lower UTS compared to Chi and LNPs-Chi films. For instance, SL_40_-Chi_60_ showed an UTS of 22 MPa compared to 91 MPa with the films containing an equal weight fraction of LNPs. As shown in Supporting Information Figure S5, the SL-Chi film showed visible particle aggregation while the LNP-Chi film appeared uniform. This fact suggests that the uniform LNP size and the enrichment of negatively charged groups on the surfaces of LNPs are important for homogeneous dispersion and restricting flocculation, coagulation, and sedimentation of lignin particles during the evaporation of the Chi matrix. It is therefore paramount to fabricate nanocomposite films with appropriate lignin particle morphology, such as the spherical ones documented in this work.

To further elucidate the effect of lignin morphology on mechanical properties, we chose the films with the largest difference in mechanical performance at similar lignin content, that is, LNPs_40_-Chi_60_ (91 MPa) and SL_40_-Chi_60_ (22 MPa), for detailed SEM characterization (Figure 3d–k). The fracture cross sections of the films revealed major differences that may explain their contrasting mechanical behavior. As shown in a higher magnification in Figure 3e,i, the fracture cross section corresponding to LNPs_40_-Chi_60_ was flat and uniform in contrast to the presence of microscopic aggregates and irregularities observed in the SL_40_-Chi_60_ specimen. The superior mechanical performance of LNPs_40_-Chi_60_ can be attributed to the lack of aggregation which would promote the fracture formation when stress is applied. In line with this reasoning, more uniform surface morphology was observed when LNPs instead of coprecipitated lignin were present in the films. SEM images show uniform surface distribution of LNPs on LNPs_40_-Chi_60_ (Supporting Information Figure S6) in contrast to the presence of micrometer sized (∼19 μm) aggregates present on the SL_40_-Chi_60_ film (Figure 3j,k). We observed uniformly distributed nanoparticles (∼119 nm) on the surface of LNPs_40_-Chi_60_ by AFM (Figure 3f,g).

### 3.3 Optical Properties of LNP-Chi Films

**Figure 4:**
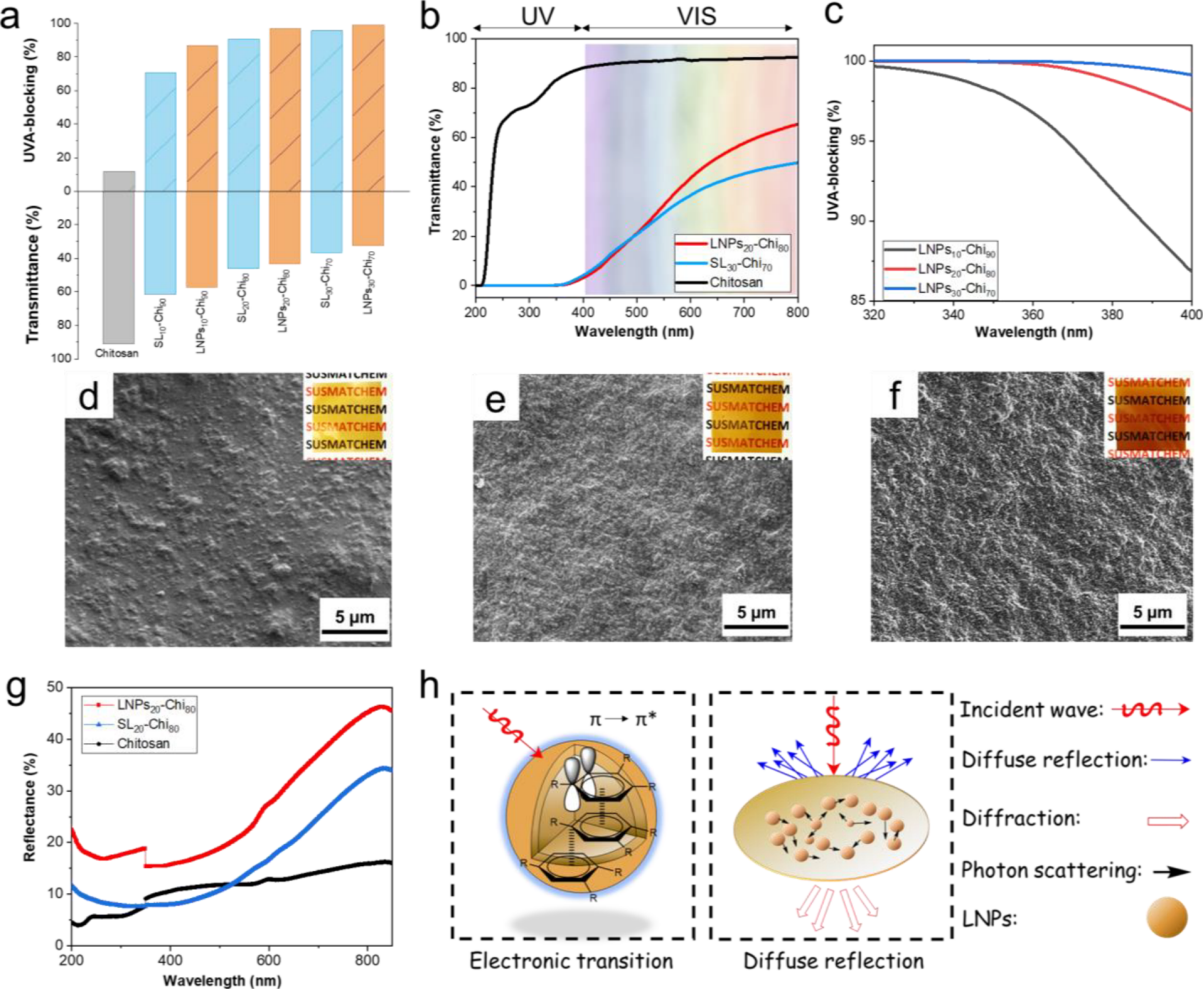
Optical properties of LNP-Chi and SL-Chi films. (a) UVA blocking and visible light transmittance of LNP-Chi and SL-Chi films were measured at 400 and 600 nm respectively. (b) In-line (direct) transmittance of LNPs_20_-Chi_80,_ SL_30_-Chi_70_, and Chi films in the UV–Vis range. (c) UVA-blocking spectra of LNP-Chi films. SEM images with corresponding digital photos of LNP-Chi films with different lignin contents (wt %), (d) LNPs_10_-Chi_90_, (e) LNPs_20_-Chi_80_, and (f) LNPs_30_-Chi_70_. (g) Reflectance spectra of LNPs_20_-Chi_80,_ SL_20_-Chi_80_, and Chi film. (h) Schematic mechanism of UV-blocking properties of LNP-Chi films. Thickness of films: ∼20 μm.

UV-blocking properties are desirable in building materials such as glass ceilings and rooftops.^1,6^ It is particularly important to block UVA radiation (wavelength at 320–400 nm), which occupies more than 95% of the total UV radiation reaching the Earth’s surface. Here, we relied on the small size and uniform distribution of LNPs in the nanocomposite films. As shown in Figure 4a, already the lowest lignin content of 10 wt % increased the UVA blocking to 87% compared to 12% with Chi alone. The highest extent of UVA blocking reached by the nanocomposite films was 99% when the film contained 30 wt % of LNPs. Regardless of the lignin morphology, there was an increasing trend of UVA blocking with increased lignin content. However, LNP-Chi films showed systematically higher extents of UVA blocking than the SL-Chi films at similar lignin contents. For example, LNPs_20_-Chi_80_ exhibited 97% UVA blocking, which was higher than that observed with SL_20_-Chi_80_ (90%) and SL_30_-Chi_70_ (96%) (Figure 4a). These results together with a clear difference in the mechanical properties (Figure 3a) rationalize and prove the selection of LNPs for the preparation of Chi-based nanocomposite films. These results add to previous knowledge on the benefits of spherical lignin particles, as recently reviewed by Österberg et al.^51^

A further advantage of LNP-Chi films is their good visible-light transmission in comparison to SL-Chi films. The UV–Vis spectra of LNPs_20_-Chi_80_ and SL_30_-Chi_70_ were selected for comparison because of their similar UVA-blocking performance. As shown in Figure 4b, LNPs_20_-Chi_80_ showed higher transmission in the visible-light range. This can be attributed to the improved uniformity of LNP-Chi films, which could also be appreciated by the visible text under the film (Figure 4e). As the weight percentage of LNPs in the films increased from 10 to 30%, the extent of UVA blocking increased from 87 to 99% (Figure 4c). There was a nonlinear correlation between the content of LNPs and the extent of UVA blocking, which suggests that 20 wt % LNPs achieves a sufficiently dense coverage in the films as also supported by the SEM images (Figure 4d–f). The optical properties of LNP-Chi films are not yet comparable to those based on synthetic polymers such as poly(ethylene bifuranoate)^58^ or PVA^59^ (Supporting Information Table S3), but here our focus is on fully biobased materials for the combination of UV-blocking and photothermal properties.

The optical properties of UV-blocking materials are highly dependent on the aromatic backbone structure^31,60^ and particle size^14,61,62^ in the material. As shown in the Fourier transform infrared (FTIR) spectrum of LNPs (Supporting Information Figure S7), aromatic skeletal vibration of lignin occurs at 1600, 1509, and 1450 cm^–1^, C–O of phenol stretching at 1207 cm^–1^, and aromatic C–H vibration out of the plane at 833 cm^–1^. The abundant aromatic rings and phenolic hydroxyl groups of lignin provide more opportunities for excitation of electrons from the highest occupied molecular orbital to lowest unoccupied molecular orbital, especially the transition from π to π*. In addition to that, the presence of conjugated structures gives rise to π–π stacking in LNPs, which contributes to their capacity to absorb light energy.^20^ Therefore, as illustrated in Figure 4h, we hypothesize that the aromatic structures and π–π stacking contribute to the efficient electronic transition from π to π* and absorption of radiation from UV light. Such electron excitations are ubiquitous in nature, for example in chloroplasts where chlorophyll acts as an electron donor. In the case of lignin, the absence of electron acceptors causes electrons to return to the ground state which releases heat.^63^ In addition to UV absorption, and although rarely distinguished in the literature, the impact of LNPs on UV blocking can be understood by reflectance measurement based on the assumption that nanoparticles have the ability to scatter the incident light. As shown in Figure 4g, LNPs_20_-Chi_80_ shows a higher reflectance in the wavelength region of 200–800 nm compared to that of the pure Chi film and SL_20_-Chi_80_. We consider that this difference in reflectance of the two films with equal lignin contents may derive from differences in their surface coverage of lignin particles. This can be attributed to the fact that nanoparticles in LNPs_20_-Chi_80_ might have suitable distribution and diameter to scatter strongly UV radiation and shorten the scattering mean free path.^64^ In summary, the radiation blocking in LNP-Chi films involves two important phenomena, electronic transition and diffuse reflection, as shown in Figure 4h.

### 3.4 Photothermal Performance of LNP-Chi Films

**Figure 5:**
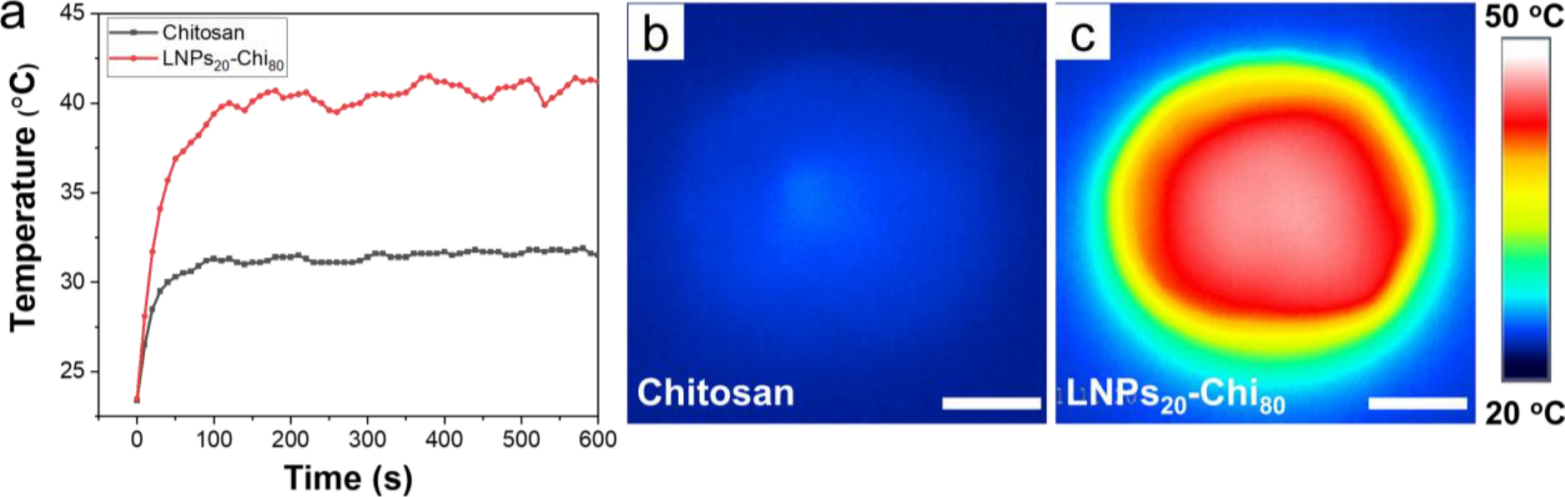
Photothermal performance of LNP-Chi films. (a) Temperature variation in LNPs_20_-Chi_80_ and Chi films under artificial solar irradiation (100 mW/cm^2^). Infrared images of irradiated (b) Chi film and (c) LNPs_20_-Chi_80_ after 10 min of exposure to simulated solar irradiation (100 mW/cm^2^); scale bar = 1.5 cm.

Having established advantageous optical properties of the LNP-Chi films, especially their efficient UV radiation adsorption, it was interesting to continue to study the photothermal performance of the films from the point of view of energy conversion. Here, we chose LNPs_20_-Chi_80_ as a demonstrator for the photothermal studies because of its combination of favorable mechanical strength and optical properties. We used an LED lamp as a light source to simulate solar radiation. As shown in Figure 5a, there was a rapid temperature increase during the initial 120 s when LNPs_20_-Chi_80_ was exposed to 1 sun (100 mW/cm^2^) irradiation. After increasing from 23 to 40 °C in 120 s, the temperature of LNPs_20_-Chi_80_ maintained around 41 °C with minor fluctuation during persistent radiation (10 min). In contrast, the Chi film only reached 31 °C under similar conditions, which indicates that LNPs play a major role in the photoenergy absorbed in the nanocomposite film. The transmission spectra of Chi and LNPs_20_-Chi_80_ films (Supporting Information Figure S8) indicate that LNPs absorb solar energy from the UV–Vis to infrared range (wavelength 200–1800 nm) more than Chi. This is evident also from the corresponding IR camera images recorded after 10 min of illumination (Figure 5b,c). These results highlight the potential of LNP-Chi nanocomposite films as promising candidates for energy-efficient buildings to modulate daylight transmittance for example in “smart” windows. Such an application would benefit from the combination of high mechanical strength and flexibility, efficient visible light transmittance, UVA blocking, and photothermal performance of these fully biobased nanocomposites.

### 3.5 Photothermal Coatings

**Figure 6:**
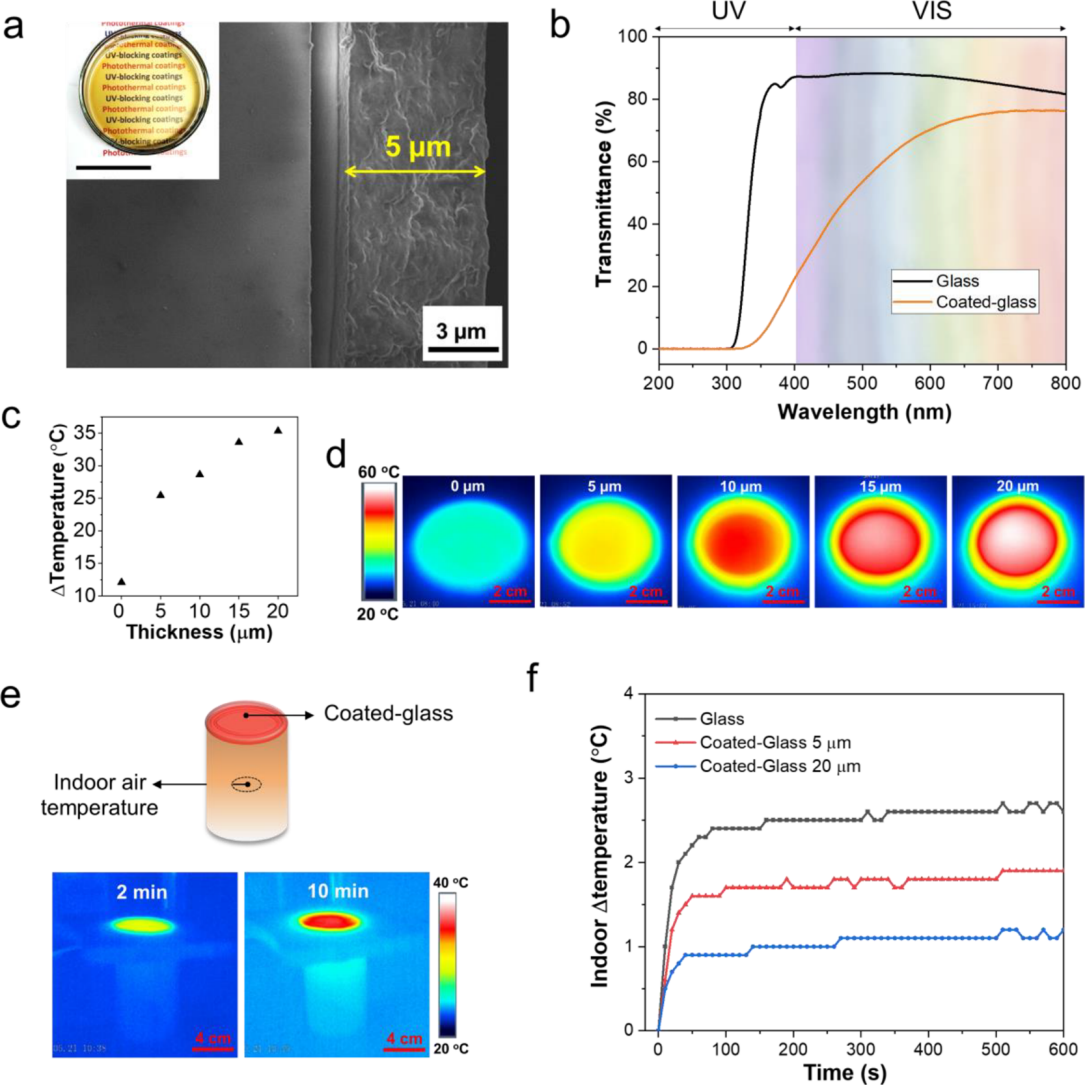
Optical and photothermal performance of LNPs_20_-Chi_80_ coated-glass with different coating thicknesses (5–20 μm). (a) SEM image of coated-glass with the corresponding digital photo (scale bar = 2.5 cm). (b) UV–Vis spectra of coated-glass (5 μm) and glass. (c) Temperature increment as a function of coating thickness. (d) Coated glass with tunable coating thickness after 10 min of irradiation under simulated solar irradiation (100 mW/cm^2^). (e) Experimental setup for measuring indoor air temperature with coated glass as a ceiling and infrared images of coated glass (20 μm) irradiated under simulated solar irradiation (100 mW/cm^2^) after 2 and 10 min. (f) Indoor air temperature change curves under simulated solar irradiation (100 mW/cm^2^) with coated glass or glass as a ceiling.

With the application of energy-efficient “smart” windows in sight, we chose glass as a substrate to apply LNPs_20_-Chi_80_ as UV-blocking and photothermal coatings. The thickness of the coating layer was assumed from the amount of dispersion deposited per area. The digital image in Figure 6a shows a transparent coating applied on glass placed over a paper with a printed text. The associated SEM image shows the glass cross section with a coating layer of 5 μm. As could be expected from its good visible light transmittance, the coated glass displayed better UV-blocking performance compared to that of pure glass, efficiently decreasing the UVA transmittance from 87 to 22% at a wavelength of 400 nm (Figure 6b). Meanwhile, the coatings did not markedly compromise light transmittance at 600 nm, with 70% transmittance compared to 87% of pure glass (Figure 6b). However, whitening industrial lignin^60^ may be needed if the yellowish brown color of the lignin coatings is undesirable in interior applications.

Having demonstrated their optical performance, we continued to measure the photothermal properties of the nanocomposite coatings. As expected from the performance of the LNPs_20_-Chi_80_ films, the coatings produced significantly increased temperature after irradiation for 10 min (Figure 6c). There was a linear correlation between increasing photothermal capacity and the coating thickness from 5 to 20 μm. As shown in Figure 6d, pure glass reached a temperature of 35 °C, while glass coated with 20 μm could be photothermally heated up to 58 °C over a similar time period. Considering real-world applications, we carried out a comparative evaluation in a simplified house model to determine changes in indoor temperature when using coated glass as a roof window (Figure 6e). From the IR camera images, one can conclude that the coating efficiently absorbed light energy, which could prevent its absorption in internal air (Figure 6e). When measured directly from the empty interior space of the house model, the coated glass (20 μm in thickness) allowed the indoor temperature to be maintained within an increment of 1.1 °C compared to 2.6 °C with uncoated glass after reaching stability at 400 s of illumination (Figure 6f). These new photothermal coatings based on abundant biobased polymers serve as green coatings to protect interior spaces from UV radiation and simultaneously restrict unwanted warming indoors and reduce the energy consumption of air conditioning.

## 4 Conclusions

We have reported the preparation of fully biobased and energy-efficient LNP-Chi nanocomposite films with lignin contents up to 40 wt %. The nanocomposites showed superior mechanical strength to films containing precipitated particles. The enhanced mechanical strength arises from the uniformly and densely packed distribution of LNPs in the chitosan matrix. Owing to their favorable radiation absorption ability, the nanocomposite films were successfully demonstrated as high-performance glass coatings for photothermal conversion and indoor temperature modulation. The coated glass showed superior UVA blocking and photothermal performance to pure glass. Finally, we hold a view that our system will encourage further efforts to develop LNPs as building blocks for multifunctional nanocomposites in photothermal applications and energy-efficient buildings.

## Supporting Information Available

Schemes of LNPs and SL-Chi film preparation, photos of films and the hybrid dispersion, ^31^P NMR spectrum of SL, FTIR spectrum of LNPs, and transmission spectra of Chi and LNPs_20_-Chi_80_ (PDF).
