# Supplementary material for: Fully Biobased Photothermal Films and Coatings for Indoor Ultraviolet Radiation and Heat Management
Source: ACS Appl Mater Interfaces. 2022 Mar 1;14(10):12693–702. doi: 10.1021/acsami.2c00718 (PMC8931727; doi:10.1021/acsami.2c00718)
Supplement: Supplementary file 1 — am2c00718_si_001.pdf [file am2c00718_si_001.pdf]

Supporting Information:

Fully bio-based photothermal films and coatings for indoor UV radiation and heat management

Jinrong Liu, [a] *Adrian Moreno,* [a] *Jian Chang,* [a] *Mohammad Morsali,* [a] *Jiayin Yuan,* [a] *Mika H. Sipponen*[a]*

[a]Department of Materials and Environmental Chemistry, Stockholm University, Svante Arrhenius väg 16C, SE-106 91 Stockholm, Sweden.

*Corresponding author: mika.sipponen@mmk.su.se

This file contains:

Supplementary Figures 1-8

Supplementary Tables 1-3

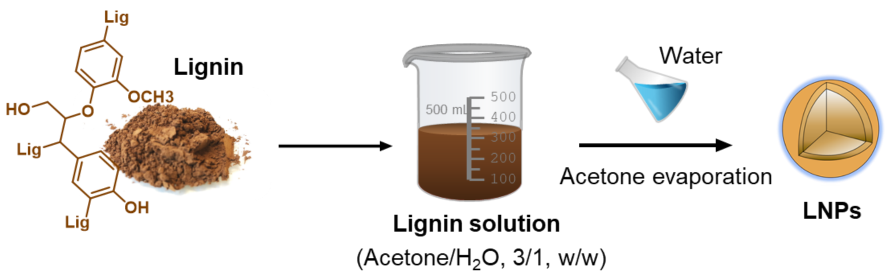

Fig S1. Scheme for the preparation of LNPs by solvent exchange method.[1]

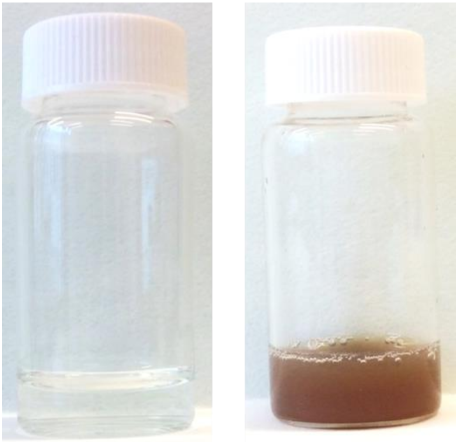

Fig S2. Photos of chitosan solution (left) and $LNPs_{20}$-Chi_{80} hybrid dispersion (right).

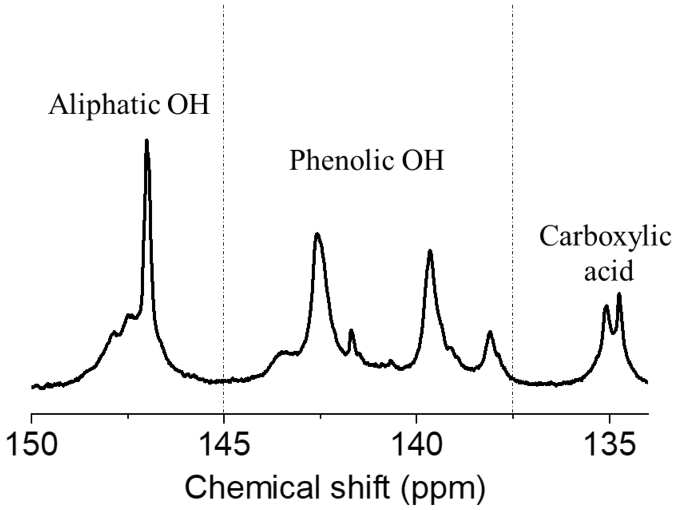

Fig S3. Quantitative ^{31}P NMR spectrum of soda lignin.

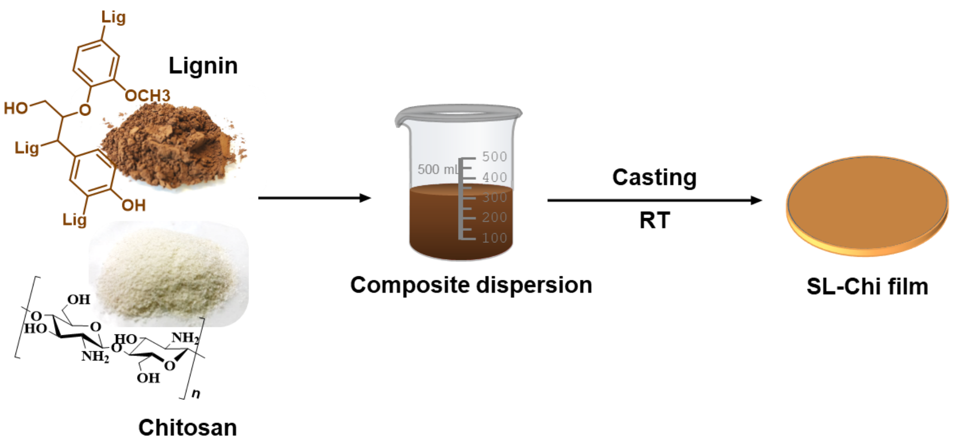

Fig S4. Scheme for the preparation of SL-Chi films.

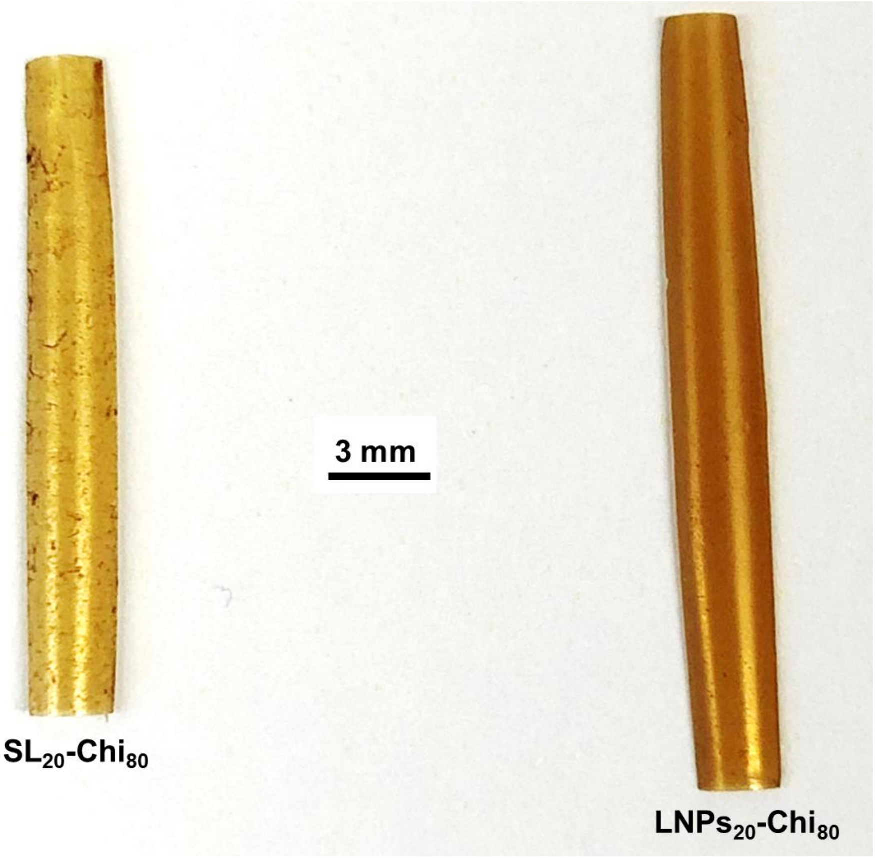

Fig S5. Photo of SL_{20}-Chi_{80} and $LNPs_{20}$-Chi_{80} films. Films were cut to rectangular shapes for tensile testing.

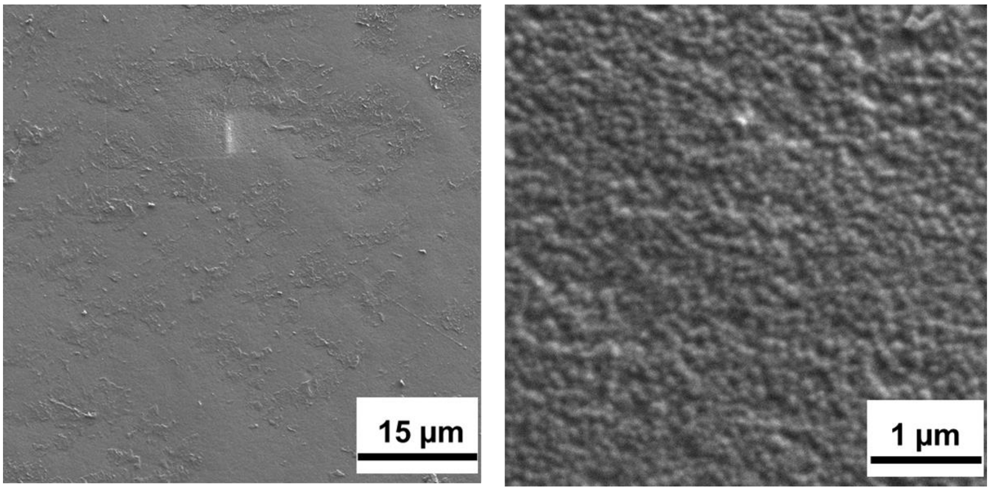

Fig S6. SEM images of the surface morphology of $LNPs_{40}$-Chi_{60}.

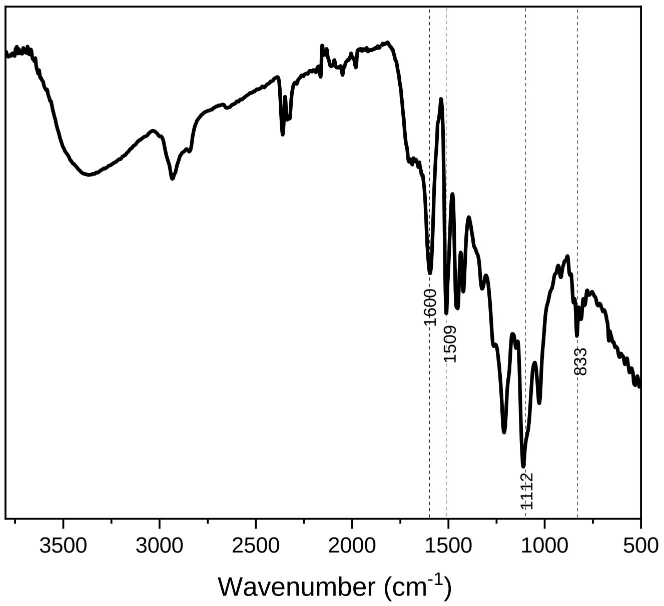

Fig S7. FTIR spectrum of LNPs.

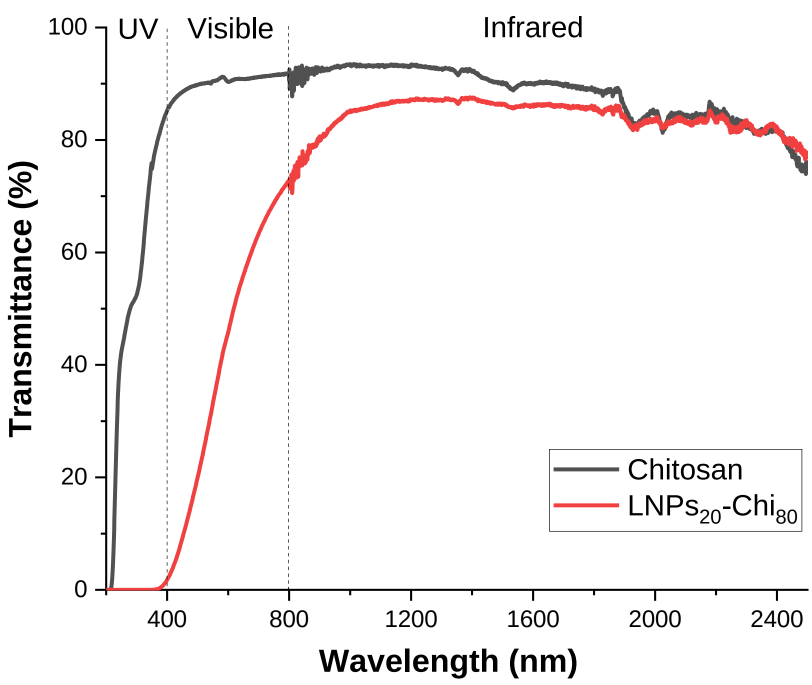

Fig S8. Transmission spectra of Chitosan and $LNPs_{20}$-Chi_{80}.

Table S1. Amount of aliphatic, phenolic, and carboxylic hydroxyls (mmol/g) in soda lignin, as determined by ^{31}P NMR.

Sample	Aliphatic OH	Phenolic OH	Carboxylic acid OH	Total OH
Soda lignin	1.34 ± 0.02	2.71 ± 0.07	0.59 ± 0.01	4.64 ± 0.10

The error bars represent one standard deviation (SD) from the mean values (n = 3).

Table S2. Ultimate tensile strength (UTS) in different materials.

Material	UTS (MPa)	Ref.
Chitosan-lignin	140	[2]
Lignin-carbohydrate	102	[3]
LNPs40-Chi60	91	This work

Table S3. UVA-blocking and Visible light transmittance properties in different materials.

	Abs-400 nm	Abs-600 nm	(Abs-400 nm)/ (Abs-600 nm)	Ref.	UVA-blocking %	Vis %
Delignified wood	0.7	0.1	7	[4]	80	78
CF +2% lignin	0.8	0.2	4	[5]	85	65
SL-10%	1.4	0.4	3.5	[6]	96	38
PBAT-LMNP-5.0%	0.9	0.5	1.8	[7]	88	34
PBMA-GOx-chi-$LNPs_5$	1.7	0.7	2.4	[1]	98	22

Poly ethylene bifuranoate	1.3	0.1	13	[8]	95	75
LA-5	2	0.3	6.7	[9]	99	50
TA@LS-Ag-5	2	0.4	5	[10]	100	43
CQDs-PVA film	1.7	0.1	17	[11]	98	80
$DS_{0.41}$	1.5	0.1	15	[12]	97	80
LNPs20-Chi80	1.5	0.4	3.7	This work	97	44

References

1. Moreno A, Morsali M, Liu J, Sipponen MH. Access to tough and transparent nanocompositesviaPickering emulsion polymerization using biocatalytic hybrid lignin nanoparticles as functional surfactants. *Green Chem*. **23**, 3001-3014 (2021).

2. Izaguirre N, Gordobil O, Robles E, Labidi J. Enhancement of UV absorbance and mechanical properties of chitosan films by the incorporation of solvolytically fractionated lignins. *Int J Biol Macromol*. **155**, 447-455 (2020).

3. Zhang F, Lan X, Peng H, Hu X, Zhao Q. A “trojan horse” camouflage strategy for high-performance cellulose paper and separators. *Adv Funct Mater*. **30**, 1-7 (2020).

4. Mi R, Chen C, Keplinger T, et al. Scalable aesthetic transparent wood for energy efficient buildings. *Nat Commun*. **11**, 1-9 (2020).

5. Sadeghifar H, Venditti R, Jur J, Gorga RE, Pawlak JJ. Cellulose-lignin biodegradable and flexible UV protection film. *ACS Sustain Chem Eng*. **5**, 625-631 (2017).

6. Xing Q, Ruch D, Dubois P, Wu L, Wang WJ. Biodegradable and High-Performance Poly(butylene adipate-co-terephthalate)-Lignin UV-Blocking Films. *ACS Sustain Chem Eng*. **5**, 10342-10351 (2017).

7. Xing Q, Buono P, Ruch D, Dubois P, Wu L, Wang WJ. Biodegradable UV-Blocking Films through Core-Shell Lignin-Melanin Nanoparticles in Poly(butylene adipate- co-terephthalate). *ACS Sustain Chem Eng*. **7**, 4147-4157 (2019).

8. Kainulainen TP, Sirviö JA, Sethi J, Hukka TI, Heiskanen JP. UV-blocking synthetic biopolymer from biomass-based bifuran diester and ethylene glycol. *Macromolecules*. **51**, 1822-1829 (2018).

9. Zhang X, Liu W, Yang D, Qiu X. Biomimetic supertough and strong biodegradable polymeric materials with improved thermal properties and excellent UV-blocking performance. *Adv Funct Mater*. **29**, 1-11 (2019).

10. Zhang X, Liu W, Sun D, et al. Very Strong, Super-Tough, Antibacterial, and Biodegradable Polymeric Materials with Excellent UV-Blocking Performance. *ChemSusChem*. **13**, 4974-4984 (2020).

11. Hess SC, Permatasari FA, Fukazawa H, et al. Direct synthesis of carbon quantum dots in aqueous polymer solution: one-pot reaction and preparation of transparent UV-blocking films. *J Mater Chem A*. **5**, 5187-5194 (2017).

12. Li B, Xu C, Liu L, Yu J, Fan Y. Facile and sustainable etherification of ethyl cellulose towards excellent UV blocking and fluorescence properties. *Green Chem*. **23**, 479-489 (2021).
